# Prefrontal Cortical Control of Activity in Nucleus Accumbens Core Is Weakened by High-Fat Diet and Prevented by Co-Treatment with N-Acetylcysteine: Implications for the Development of Obesity

**DOI:** 10.3390/ijms231710089

**Published:** 2022-09-03

**Authors:** Carlos Morgan, Patricio Sáez-Briones, Rafael Barra, Andrea Reyes, Katherine Zepeda-Morales, Luis Constandil, Miguel Ríos, Paulina Ramírez, Héctor Burgos, Alejandro Hernández

**Affiliations:** 1 Laboratorio de Neurofarmacología y Comportamiento, Escuela de Medicina, Facultad de Ciencias Médicas, Universidad de Santiago de Chile, Santiago 9170022, Chile; 2 Centro de Investigación Biomédica y Aplicada (CIBAP), Escuela de Medicina, Facultad de Ciencias Médicas, Universidad de Santiago de Chile, Santiago 9170022, Chile; 3 Laboratorio de Neurobiología, Facultad de Química y Biología, Universidad de Santiago de Chile, Santiago 9170022, Chile; 4 Bluestone Center for Clinical Research, Department of Oral and Maxillofacial Surgery, New York University College of Dentistry, New York, NY 10010, USA; 5 Escuela de Psicología, Facultad de Medicina y Ciencias de la Salud, Universidad Mayor, Santiago 7570008, Chile

**Keywords:** ΔFosB expression, high-fat diet, N-acetylcysteine, nucleus accumbens, long-term depression, medial prefrontal cortex, mouse

## Abstract

A loss of neuroplastic control on nucleus accumbens (NAc) neuronal activity exerted by the medial prefrontal cortex (mPFC) through long-term depression (LTD) is involved in triggering drug-seeking behavior and relapse on several substances of abuse due to impaired glutamate homeostasis in tripartite synapses of the nucleus accumbens (NAc) core. To test whether this maladaptive neuroplastic mechanism underlies the addiction-like behavior induced in young mice by a high-fat diet (HFD), we utilized 28-days-old male mice fed HFD ad-libitum over 2 weeks, followed by 5 days of HFD abstinence. Control groups were fed a regular diet. HFD fed mice showed increased ΔFosB levels in the NAc core region, whereas LTD triggered from the mPFC became suppressed. Interestingly, LTD suppression was prevented by an i.p. injection of 100 mg/kg N-acetylcysteine 2.5 h before inducing LTD from the mPFC. In addition, excessive weight gain due to HFD feeding was diminished by adding 2mg/mL N-acetylcysteine in drinking water. Those results show a loss of neuroplastic mPFC control over NAc core activity induced by HFD consumption in young subjects. In conclusion, *ad libitum* consumption of HFD can lead to neuroplastic changes an addiction-like behavior that can be prevented by N-acetylcysteine, helping to decrease the rate of excessive weight gain.

## 1. Introduction

Western lifestyles have a deep impact in promoting obesity. They provide an abundance of calories during the whole year, allowing wide consumption of energy-dense foods, highly palatable diets and refined sugars, but involving a scarcity of vegetables and fruits; together with this, there are disruptions of the dark–light and sleep–awake cycles and a lack of time for leisure and for energy expenditure [1,2]. Palatable food (i.e., high-fat and high-sugar foods) intake, as an escalating addictive process leading to obesity, has been proposed by several authors (for a recent review see O’Connor and Kenny [3]).

The available data suggest that repeated sucrose access leads to upregulation of dopamine release [4] and dopamine transporters [5] in the nucleus accumbens (NAc), together with escalation of sucrose bingeing and enhanced anxiety as withdrawal symptoms [4]. Concerning high-fat foods, it has been reported that addiction-like states may emerge in adult rat bingeing on a high-fat diet (HFD) [6] and in adult mice after early exposure to ahigh-fat diet [7], which affects the NAc dopamine and enkephalin systems in a similar way to that observed in drugs of abuse [8,9]. Repeated access to high-fat food in young mice generates preference for HFD in an NMDA-receptor-dependent manner [10] and enhances dopamine release [11] in the NAc. Additionally, withdrawal from a high-fat diet, but not from a low-fat diet, potentiates anxiety and basal corticosterone levels and generates enhanced motivation for high-fat food rewards in mice [12].

The development of drug addiction entails an impaired ability of the corticostriatal projecting system to control drug-seeking behavior, a circuit that arises mainly from the medial prefrontal cortex (mPFC) and projects onto the NAc. This nucleus serves as a gateway through which information processed in the limbic system gains access to motor systems responsible for drug seeking. Self-administration of addictive drugs in rodents (e.g., cocaine) has been found to result in enduring impairment in glutamatergic mPFC-NAc pathways in terms of a long-term potentiation (LTP)-like increase in the AMPA/NMDA receptors ratio occurring in the NAc during drug abstinence [13], while NAc neurons develop an inability to elicit long-term depression (LTD) [14,15,16]. Consistently, it has been reported that rats treated with chronic cocaine displayed significant induction of ΔFosB (a truncated product of the *FosB* gene) in spiny neurons of the NAc [17]. Very similar to cocaine administration, obesity induced in adult rats by a high-sucrose, high-lipid ‘western’ diet suppressed LTD and led to greater potentiation of PFC-to-NAc synapses, as measured by AMPA/NMDA currents in slice preparations containing the NAc [18]. In addition, it was reported that HFD increased ΔFosB expression levels in NAc neurons of adult mice [12,19]. It is then apparent that the preference for HFD is a decision-making task based on reward and should be reflected in neuroplastic changes in the mPFC-NAc pathway, as seen by Peter Kalivas laboratory in all cocaine- [15], heroin- [20] and nicotine- seeking [21] behavior and relapse.

The mPFC is crucial in the top-down regulation of cognitive flexibility, decision-making and inhibitory control [22,23,24,25,26], and is also relevant to human fear learning and conditioning [27,28]. In the mPFC, both prelimbic and infralimbic regions contribute to the inhibitory control of cocaine-seeking behavior [29]. In vivo optogenetic prelimbic mPFC stimulation significantly prevented compulsive cocaine seeking in rats, whereas optogenetic prelimbic cortex inhibition significantly increased compulsive cocaine seeking [30]. Therefore, it is apparent that either the inhibition of glutamatergic neurons projecting from mPFC to the NAc [31] or the inability of NAc neurons to develop LTD in response to glutamate input from mPFC [15,18] promotes the behavioral sensitization phenotype as found in animals previously primed either with an addictive drug or a palatable diet, leading to a poor cortical control of NAc activity. These observations have been mostly confirmed further in humans, using transcranial magnetic stimulation of the prefrontal cortex to reduce both cocaine use and craving (for a recent review see Torres-Castaño et al. [32]).

Recent advances concerning the neuronal mechanisms by which cannabinoids exert their orexigenic effects have shed some light on the role of specific cortical regions and circuitries in the development of food addiction-like behavior in mice and humans [31,33,34]. Based on the observation that brain glutamatergic activity inhibits eating behavior while GABAergic activity promotes it, Bellochio et al. [31] described a bimodal action of cannabinoids on food intake by two neuronal populations that express the CB1 receptor: the glutamatergic neurons of the dorsal telencephalon and the ventrostriatal GABAergic neurons. Conditional knockout mice for CB1 in any and both populations (Glu-CB1^−/−^ and GABA-CB1^−/−^, respectively) showed similar food intake levels for Glu-CB1^−/−^ mice and wildtype littermates, while GABA-CB1^−/−^ mice were hyperphagic. CB1 receptors in glutamatergic neurons control glutamatergic activity, accounting for some orexigenic effects of cannabinoids, and CB1 receptors in GABAergic forebrain neurons reduce the inhibitory transmission on stimulated food intake [31]. More recently, Glu-CB1^−/−^ mice have been shown to be protected from developing food addiction-like behavior, with a concomitant high excitatory transmission in mPFC and NAc [33]. Moreover, chemogenetic inhibition of the mPFC-NAc pathway resulted in compulsive food seeking and consumption of palatable foods [34], highlighting a role for glutamatergic the mPFC-NAc pathway.

Since dysregulated eating behaviors begin to emerge early in life, during childhood and adolescence [35,36], in the current study we examined the ability of HFD-priming of young Swiss-CD1 mice to worsen neuroplastic indices in the mPFC-NAc core pathway and develop obesity in those animals. As recently reported, preference for HFD is not a spontaneous behavior in Swiss CD1 mice, but it can be acquired after a priming experience of consuming HFD *ad libitum* for 2 weeks when they are young [10]. Modifications in neuroplasticity were assessed by testing changes in the ability of mPFC-NAc core synapses to develop LTD in vivo, and through modification in the expression of ΔFosB in the NAc core. Since cocaine relapse can be prevented in the rat by restoring the neuroplastic capacity of the mPFC-NAc pathway by administering N-acetylcysteine [15], we also studied the possibility that N-acetylcysteine could restore the ability of mPFC-NAc synapses to develop LTD in NAc core neurons of HFD-primed young mice, thereby protecting against obesity derived from HFD preference. N-acetylcysteine is a molecule that can reinstate glutamate homeostasis in the mPFC-NAc tripartite synapses [37], and has been proposed for preventing both cocaine relapse in animals [38] and craving in cocaine-quitters [39]. Additionally, it has also been reported that N-acetylcysteine can decrease binge eating in adult rats subjected to a high-fat, high-carbohydrate western diet [40].

## 2. Results

**Figure 1:**
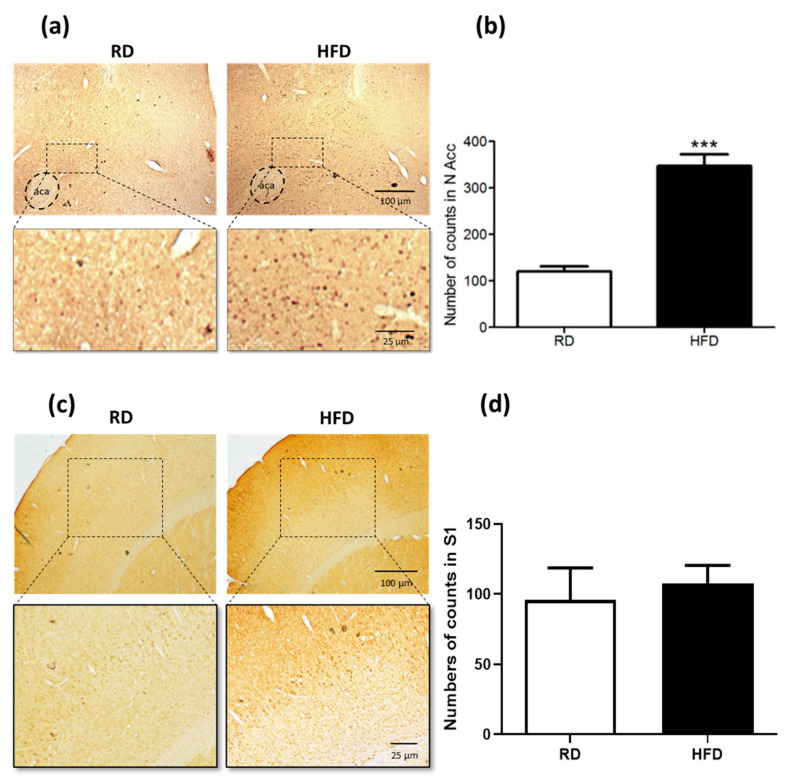
ΔFos B expression in NAc core and somatosensory S1 cortex of young mice fed either RD or HFD for 2 weeks and then subjected to an abstinence period for 5 days. (**a**) Upper panels are 500 µm wide × 400 µm height photomicrographs of 30-µm thick brain coronal sections passing at the level of the NAc (about 1.4 mm ahead bregma), taken from the left hemisphere of an RD-fed mouse (left) and a HFD-primed mouse (right), showing ΔFosB immunoreactivity. A rectangular area in the photomicrographs (enclosed by dashed line) is shown in the lower panels, magnified ×4 concerning the upper ones (see scale bars). RD: regular diet; HFD: high-fat diet). aca: anterior commissure, anterior limb. Positive ΔFosB immunoreactivity is mainly observed in formerly HFD-fed animals as small brown spots. (**b**) Count of neurons showing immunoreactivity for ΔFosB in both the left and right NAc cores (NAcc) from 6 RD-fed and 6 HFD-fed mice. The counts in the left and right sections were added together. Values are the mean ± SEM. A comparison of data from RD-fed and HFD-fed mice was made using a two-tailed unpaired Student’s *t*-test (*** *p* < 0.001, *t* = 17.49). (**c**) Upper panels are 500 µm wide × 400 µm height photomicrographs of 30-µm thick brain coronal sections passing through the somatosensory S1 cortex (about −2.0 mm behind bregma), taken from the left hemisphere of an RD-fed mouse (**left**) and a HFD-primed mouse (**right**), showing ΔFosB immunoreactivity. A rectangular area in the photomicrographs (enclosed by dashed line) is show in the lower panels, magnified ×3 concerning the upper ones (see scale bars). RD: regular diet; HFD: high-fat diet). (**d**) Count of neurons showing immunoreactivity for ΔFosB in both the left and right S1 cortex from 6 RD-fed and 6 HFD-fed mice. The counts in the left and right sections were added together. Values are the mean ± SEM. A comparison of data from RD-fed and HFD-fed mice was made using a two-tailed unpaired Student’s *t*-test (*p* = 0.3776, *t* = 0.162, not significant).

First, Figure 1a shows that ΔFosB immunoreactivity increased in NAc core from HFD-primed mice after 5 days of abstinence, compared to NAc core from RD control mice, the difference being statistically significant (*p* < 0.001, two-tailed unpaired Student’s *t*-test). Upregulated ΔFosB levels after HFD consumption had previously been observed in the NAc of adult mice [12,19] or in the NAc of mouse overexpressing ΔFosB [41], but not yet in young mice after such a short HFD-priming period (2 weeks). No significant difference in ΔFosB immunoreactivity was found when the S1 cortices from HFD-primed mice and RD control mice were compared to each other (Figure 1c,d), thus suggesting that the ΔFosB upregulation after chronic HFD consumption is rather selective for some brain regions as the NAc core.

**Figure 2:**
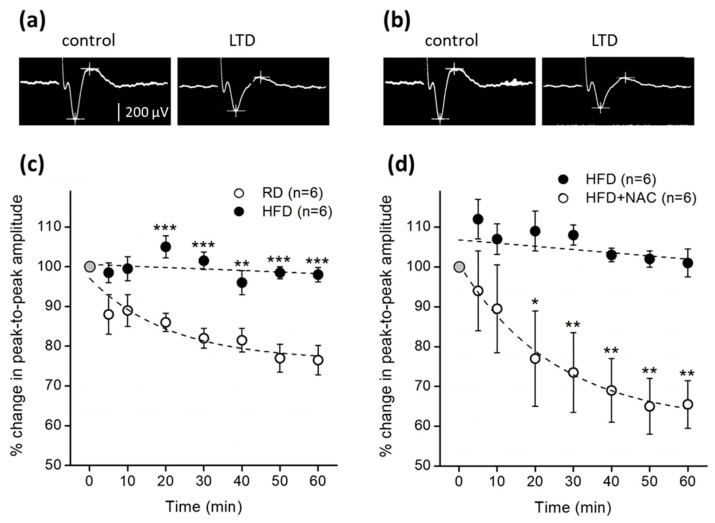
Characterization of in vivo LTD in the NAc core of young mice fed either RD or HFD for 2 weeks and then subjected to an abstinence period for 5 days. Field potentials were evoked in the NAc core from the ipsilateral mPFC and the effect of an LTD-evoking protocol assessed. (**a**) Representative example of the average of 12 successive mPFC-NAc core evoked field responses in a mouse fed RD, before (control) and after (LTD) application of the LTD- evoking protocol (see Materials and Methods); peak-to-peak amplitude is indicated by cursors (see calibration bar). The LTD-evoking protocol failed to evoke LTD in HFD-primed mice (not shown). (**b**) Similar to (**a**), but this time field responses were evoked in NAc of a HFD-primed pre-treated with 100 mg/kg i.p. N-acetylcysteine 2.5 h before the application of the LTD protocol. Note that N-acetylcysteine prevented the LTD from failing. (**c**) Time-course of % change in peak-to-peak amplitude of field potential evoked in the NAc core by electrical stimulation of the ipsilateral mPFC, after application of the low-frequency stimulation protocol at time zero min. Two-way repeated measures ANOVA revealed a treatment effect (RD vs. HFD, n = 6 mice per group, F_1, 10_ = 95.77). Asterisks indicate statistically significant intergroup differences (** *p* < 0.01, *** *p* < 0.001, Bonferroni multiple comparisons *post-hoc* test), as the LTD normally evoked by the low-frequency stimulation protocol in the NAc core of control mice fed RD (empty circles), was suppressed in the NAC core of HFD-primed mice as revealed by unchanged peak-to-peak amplitude of NAc core responses (filled circles). (**d**) 100 mg/kg i.p. N-acetylcysteine, administered 2.5 h before the application of the low-frequency stimulation protocol, prevented the LTD suppression in HDF-primed mice. Two-way repeated measures ANOVA revealed a treatment effect (HFD vs. HFD+NAC, n = 6 mice per group, F_1, 10_ = 56.22). Asterisks indicate statistically significant intergroup differences (* *p* < 0.05, ** *p* < 0.01, Bonferroni multiple comparisons *post-hoc* test), as N-acetylcysteine prevented LTD suppression in HFD-primed mice.

Second, we observed that LTD evoked in NAc core by the 3-train stimulation protocol applied into the mPFC could be observed in mice fed RD but not in mice fed HFD, the difference being statistically significant (** *p* < 0.01, *** *p* < 0.001, two-way repeated measures ANOVA followed by Bonferroni multiple comparisons test) (Figure 2a). N-acetylcysteine administered 2.5 h prior to electrophysiology prevented the LTD suppression observed in HDF-primed mice without N-acetylcysteine treatment (* *p* < 0.05, ** *p* < 0.01, two-way repeated measures ANOVA followed by Bonferroni multiple comparisons test) (Figure 2b). Interestingly, adult rats becoming obese by eating a high-sucrose, high-lipid ‘western’ diet for 8 weeks have also been reported to lack LTD in the NAc [18].

**Figure 3:**
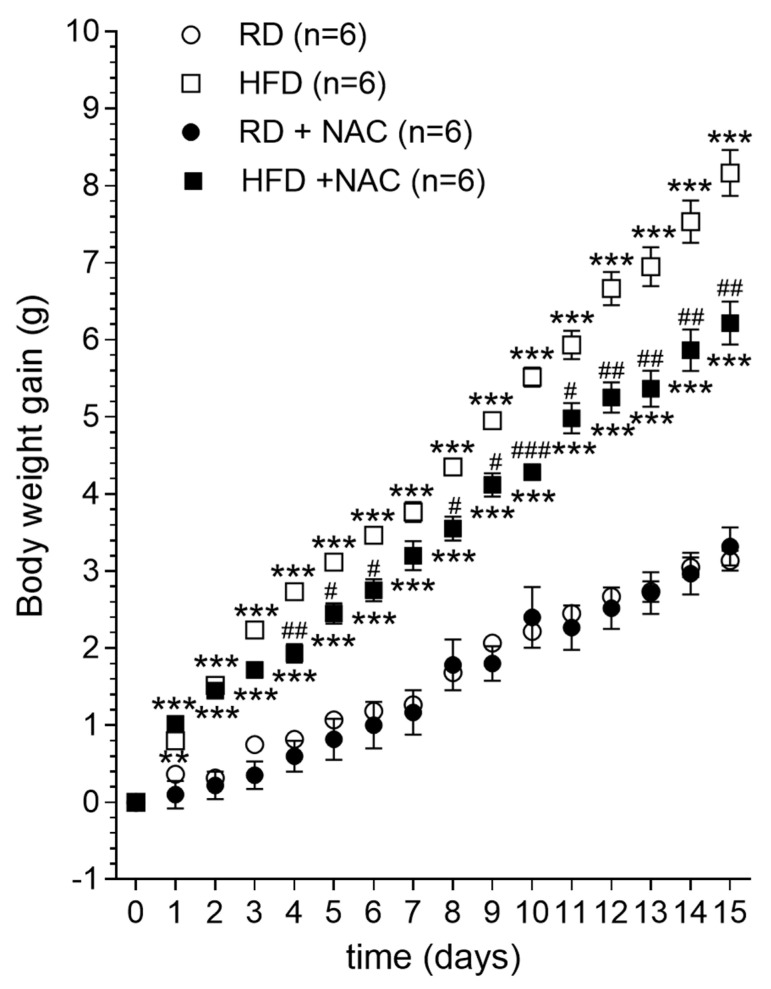
Time-course of body weight gain in young mice fed either RD or HFD for 2 weeks, and the effect of 2g/L N-acetylcysteine co-administered in drinking water. RD: regular diet; HFD: high-fat diet; NAC: N-acetylcysteine. Two-way repeated measures ANOVA revealed an intergroup effect of treatments (n = 6 mice per group, F_3, 20_ = 89.39). Mice fed HFD (empty squares) had statistically higher body weight gain than those mice fed RD (empty circles) (** *p* < 0.01, *** *p* < 0.001, Bonferroni multiple comparisons *post-hoc* test). N-acetylcysteine co-administered in drinking water resulted in a significant decrease in body weight gain of animals being feeding with HFD (filled squares) as compared to HFD-primed mice without N-acetylcysteine co-treatment (**^#^** *p* < 0.05, **^##^** *p* < 0.01, **^###^** *p* < 0.001, two-way repeated measures ANOVA followed by Bonferroni multiple comparisons *post-hoc* test), although N-acetylcysteine-treated HFD-primed mice continued to be overweight compared to control RD mice, as revealed by body weight gain curves (*** *p* < 0.001, two-way repeated measures ANOVA followed by Bonferroni multiple comparisons *post-hoc* test). Co-administration of N-acetylcysteine did not modify body weight gain in RD-fed mice.

Finally, an approach like this should be useful only if HFD-primed subjects effectively show decreased body weight gain when co-treated with N-acetylcysteine. In this regard, the results (Figure 3) show a significantly higher body weight gain in mice fed HFD versus those fed RD (*** *p* < 0.001, two-way repeated measures ANOVA followed by Bonferroni multiple comparisons test). Results also showed a significant decrease in the body weight of animals that received N-acetylcysteine in their drinking water along with being fed with HFD (**^#^** *p* < 0.05, **^##^** *p* < 0.01, two-way repeated measures ANOVA followed by Bonferroni multiple comparisons test), although those mice continued to be overweight compared to control RD mice, as revealed by body weight gain (*** *p* < 0.001, two-way repeated measures ANOVA followed by Bonferroni multiple comparisons test) (Figure 3).

## 3. Discussion

The results showed that HFD-priming for a short 2-week period in young mice, followed by 5 days of HFD abstinence, resulted in upregulated levels of ΔFosB in the NAc core, altogether with suppression of LTD triggered in the NAcc from the PFC. N-acetylcysteine administered 2.5 h prior to electrophysiology prevented LTD suppression observed in HFD-primed mice. Finally, as expected, body weight of HFD-primed mice was significantly higher than those fed RD, but this effect of HFD feeding on body weight was partially counteracted by co-administration of N-acetylcysteine.

Elevated levels of ΔFosB have been previously observed in the NAc of adult mice after prolonged HFD consumption [12,19] and in the NAc of ΔFosB-overexpressing adult mice that fed HFD when they were young [41]. ΔFosB, a sustained molecular switch that accumulates within a subset of neurons of the nucleus accumbens during addiction, likely contributes to the long-term neural and behavioral plasticity that underlies addiction [42], and can be related to the LTP-like AMPA/NMDA ratio increase occurring in the NAc during seeking behavior following cocaine withdrawal [43].

The increased ΔFosB expression observed here may be a consequence of the inability of mPFC-NAc core synapses to develop the LTD we found in HFD-primed mice, and therefore to control the activity of spiny neurons in the NAc core, a behavior closely similar to the loss of LTD reported in the NAc of rodents subjected to withdrawal from cocaine [14,15,16,44], heroin [20] and alcohol [45,46]. This finding is also comparable to the ΔFosB accumulation found in the NAc of animals subjected to repeated cocaine administration [47], especially in spiny neurons coexpressing the dopamine D1 receptor [17,48]. LTD suppression observed in animals submitted to chronic cocaine has been proposed to be the result of a metaplasticity phenomenon which inhibits further induction of synaptic plasticity in the NAc core [15]. Although the neurobiological bases for such a metaplastic process are still unclear, this may well be based—among other factors—in the changes already reported in AMPA/NMDA receptor-mediated excitatory postsynaptic currents showing NAc slices prepared from animals displaying behavioral sensitization after repeated in vivo cocaine exposure [49]. In this regard, more recent results indicate that AMPA/NMDA receptor ratio is decreased in spiny neurons of the Nac of rats experiencing sucrose craving [50] and in spiny neurons of the dorsal striatum of mice rendered on a high-fat, high-sugar ‘western’ diet [51], which suggests a decreased glutamate-mediated synaptic strength and neurotransmission in the prefrontal cortical/NAc communication. The fact that chemogenetic silencing of mPFC neurons projecting to the NAc core enhanced specifically the compulsive eating behavior, triggering animals to be unable to stop consuming palatable food despite negative consequences [41], points to glutamate availability in the mPFC-NAc synapses being a crucial factor to determining the behavioral sensitization phenotype as found in animals previously primed either with a palatable diet, heroin or cocaine [9]. Indeed, as noted elsewhere [52], metaplasticity (e.g., LTP enhancement and/or LTD suppression in the NAc) is related to how afferent activity (from the mPFC) is presynaptically translated into neurotransmitter release and how glutamate release is postsynaptically coupled to receptor activation and intracellular calcium signaling in the NAc. Translated to the mPFC-NAc core pathway, this confers mPFC the ability to play a crucial role in the transition to and the persistence of the addictive behavior [24,25] as part of its more general role in top-down regulation of cognitive flexibility, decision-making and inhibitory control [22].

In our setup, mPFC could not evoke an LTD on NAc in HFD feeding mice, suggesting that mPFC had, at least in part, lost its ability to control NAc activity. The fact that N-acetylcysteine could rescue the LTD in NAc core glutamatergic synapses of HFD-primed mice can be explained by the glutamate homeostatic hypothesis on drug addiction [37,52]. This hypothesis proposes that reduction of mGluR2/3 and mGluR5 metabotropic receptor stimulation in the NAc, caused by failure of the cystine-glutamate antiporter in providing adequate amounts of extrasynaptic glutamate from astrocytes, is involved in drug seeking and reward. N-acetylcysteine would correct this situation by restoring extra-synaptic glutamate levels and thereby the glutamate-mediated synaptic strength and neurotransmission in the prefrontal cortical/Nac core communication. N-acetylcysteine forms cysteine in the blood circulation which enters the blood-brain barrier and is rapidly oxidized to cystine, which is in turn exchanged by glutamate in glial cells. This approach has been shown to be useful in restoring extra-synaptic glutamate levels provided by glial expression of the antiporter protein, Xc, and the glutamate transporter, GLT-1. The present in vivo observation that a single injection of N-Acetylcysteine can restore the synaptic plasticity is promising as a therapeutic target to recover obese patients from their “food addiction”.

Finally, significant differences were found in body weight gain of HFD-primed young mice treated and non-treated with N-acetylcysteine co-administered in drinking water. As shown in the Results section, the body weight of HFD-primed mice was significantly higher than those feeding RD, an effect that was partially but significantly counteracted by the co-administration of N-acetylcysteine. Similar results were previously reported by Ma et al. [53], who treated adult mice with N-acetylcysteine for 11 weeks. In contrast, the current study involved young mice treated for only 2 weeks. Importantly, Ma et al. [53] found that 11 weeks N-acetylcysteine treatment in HFD feeding mice diminished chronic inflammation in adipose tissue, improved obesity-associated insulin resistance and blocked lipid accumulation in the liver, altogether with increasing the expression of some genes involved in energy expenditure [53]. Hence, the data of Ma et al. [53] and the present results provide evidence pointing to N-acetylcysteine supplementation as a means of blocking diet-induced obesity and associated comorbidities. In this regard, N-acetylcysteine treatment has been found to reduce cocaine seeking in rodents and craving in cocaine-dependent humans [54]. Medical case reports suggest that N-acetylcysteine could function as a synergist to improve binge eating symptoms and pathological hair plucking [55], as well as treat some repetitive obsessive-compulsive-related disorders (skin-picking disorders, trichotillomania, and nail biting) [56], but systematic studies in clinical settings aimed at treating eating disorders with N-acetylcysteine have not yet been conducted.

In the context of the Koob and Volkow’s addiction model [34], the observed LTD suppression most likely reflects a weakened ability of mPFC to control addictive behavior through the mPFC-NAc pathway; however, bingeing, craving and relapsing on the HFD addiction cycle needs further characterization.

As a whole, the above results showed that food addiction is based on a maladaptive neuroplasticity, which may account for a notable portion of the obesity epidemic [57]. The present study gave rise to positive results in favor of N-Acetylcysteine treatment of high-fat diet addiction-like symptoms, a syndrome that can be observed in people as it is being investigated in the medical area [58]. N-Acetylcysteine has been used in some limited trials with promising results in treating people with cocaine-dependence [59], but the clinical efficacy of NAC in substance use disorders has not been fully established [60].

Whether highly palatable food, including those comprising high sugar and high fat diets, may be considered substances of abuse and dependence awaits further investigations. Food addiction as a concept emerged operationally and is currently considered as a process addiction, similar to binge-eating disorder, pathological gambling, compulsive buying and other behavioral addictive disorders. Both drug and non-drug addictions, namely process addictions, share common domains based on similar brain mechanisms regarding reward deficit, stress sensitization and impaired inhibitory control (Koob & Volkow, 2016) [34].

## 4. Materials and Methods

### 4.1. Animals and Diets

The experiments were performed in young Swiss CD1 male mice from an inbred colony derived from mice purchased to Charles River Laboratories, Worcester, MA, USA. Mice that were 4 weeks-old were fed either a regular diet (RD) or a HFD (Research Diets, New Brunswick, NJ, USA) for 2 weeks, according to Butiggieg et al. [10]; during this period, all animals were controlled daily for body weight. Then, HFD-fed mice were switched to RD for 5 days in order to commence HFD abstinence, allowing the development of craving for fatty foods, while RD-fed mice continued on their same diet. Thereafter, the animals were assigned either to electrophysiology or immunohistochemistry. Mice assigned to electrophysiology and immunohistochemistry were different animals, because the stimulation paradigm used to induce LTD might eventually modify the expression level of ΔFosB in the NAc [17]. The experimental protocols and animal management followed the NIH Guide for the Care and Use of Laboratory Animals [61] and were approved by the Institutional Ethics Committee of the University of Santiago de Chile (protocol #04/2019).

### 4.2. Experiment 1: FosB/ΔFosB Immunohistochemistry in NAcc and S1 Cortex

This experiment was designed to assess the ability of a HFD, followed by 5 days of HFD withdrawal, to generate increased neuronal activity in the NAc core of young mice (as revealed by increased expression levels of ΔFosB, since because previous reports associating upregulated levels of ΔFosB to HFD consumption were performed on adult mice [12,19] or in the ΔFosB overexpressing mouse [41]). In total, 6 RD-fed and 6 HFD-fed mice were used in this experiment.

Mice were anaesthetized with 30% urethane and intracardially perfused with phosphate buffered saline (PBS), followed by 4% paraformaldehyde in PBS with 2% picric acid. Brains were removed and post-fixed in 4% paraformaldehyde for 24 h and cryopreserved in 30% sucrose; then, 30 µm coronal sections were cut on a microtome. FosB-ΔFosB immunoreactivity was carried out in free-floating sections. Before blocking with 3% normal goat serum in 0,1M Phosphate Buffer pH7,4 (PB) for 2 h, sections were rinsed with methanol and 0.3% hydrogen peroxide for 30 min. After blocking, sections were incubated overnight at room temperature with primary antibody (rabbit polyclonal anti FosB and isoforms of ΔFosB dilution 1:500 in PB; sc-48, Santa Cruz Biotechnology, Santa Cruz, CA, USA). Then, sections were rinsed with PB and incubated for 1.5 h at room temperature in biotinylated secondary antibody (goat anti-rabbit IgG dilution 1:200 in PB, Jackson Immuno Research, West Grove, PA, USA). A biotin-streptavidin technique was used to visualize FosB-ΔFosB immunoreactivity (Kit ABC Elite Vectastain, Vector, San Diego, CA, USA), and was used according to the manufacturer’s instruction; 0.2% 3,3′diaminobenzidine was used as the chromagen.

Changes in FosB-ΔFosB expression levels induced by the treatments were analyzed in coronal brain sections containing the nucleus accumbens core, or the somatosensory cortex that served as a control. Quantitative measurement was performed counting FosB-ΔFosB immunoreactive spots (FosB-ΔFosB ir) on sections from both right and left hemispheres. For each mouse, the boundaries of the left and right NAc core regions were delineated on a cresyl violet-stained coronal brain section passing approximately 1.4 mm anterior to the bregma, and a bilateral manual count of ΔFosB-immunoreactive cells was performed under camera lucida within the outlined boundaries of both NAc core using an adjacent coronal section. Manual counting of ΔFosB-immunoreactive cells in the S1 cortex, a relatively large region, was confined to a 500 µm × 400 µm rectangular area outlined within a coronal section of the brain that passed approximately −2.0 mm behind bregma. Only immune-positive cells with clearly defined boundaries were counted, regardless of cell size or shape. The number of neurons identified to express ΔFosB was obtained by averaging the number of ΔFosB-expressing neurons in the sections obtained from 6 RD-fed and 6 HFD-fed mice. Bright-field images were obtained with an Olympus BX53 microscope and a Micropublisher 3.3RTV camera using a magnification of 10X. FIJI (Image J) software was used to adjust contrast and brightness.

### 4.3. Experiment 2: In Vivo Assessment of LTD in the NAcc and Effect of N-Acetylcysteine

The aim of this experimental series was to assess, by means of in vivo electrophysiology, the effect of HFD and N-acetylcysteine on LTD. Five days after HFD-fed mice were switched to RD (mice fed RD continued their diet), half of HFD-primed or RD animals received a single i.p. dose of 100 mg/kg N-acetylcysteine dissolved in saline, 2.5 h before initiating basal recordings of field potentials from the Nacc, according to the protocol of Moussawi et al. [15], while the other half of animals received i.p. the solvent alone. In total, 12 RD-fed and 12 HFD-fed mice were used in this experiment.

Mice were anesthetized with 1.5 g/kg urethane and placed in a stereotaxic frame. A stimulating bipolar electrode (two side-by-side glued 50-µm-diameter insulated tungsten wires with a 0.5-mm tip separation) was inserted into the left mPFC, at coordinates A = 1.7 and L = 0.6 regarding Bregma, V = −2.0 mm from the cortical surface. The monopolar recording electrode (a 20-µm tip diameter tungsten semi-microelectrode) was inserted into the ipsilateral NAc core at coordinates, A = 1.7; L = 1.3; V = 4.5 mm from bregma. For testing stimulation, rectangular electric pulses 0.2 ms in duration and 0.2 Hz in frequency, generated by a Grass S11 stimulator in conjunction with a Grass SIU-5 stimulus isolation unit and a Grass CCU 1A constant current unit, were applied in the mPFC through the stimulating bipolar electrode with the necessary current intensity to obtain NAc core responses of 500 µV peak-to-peak. LTD was evoked in vivo in the Nac core by applying 3 trains of 900 pulses in the mPFC with 1.5-fold of the current intensity required for testing, at 5 Hz for 3 min each one with 5 min inter-train interval, according to Moussawi et al. [15]. Recordings were amplified by a Grass P-511 preamplifier (0.8–1000 Hz bandwidth), displayed on a Philips PM 3365A digital oscilloscope, digitized at a rate of 10,000/s by an A/D converter interfaced to a computer, and stored for retrieval and off-line analysis. The efficacy of the stimulating trains to depress field responses evoked in the NAc core was evaluated by measuring the peak-to-peak amplitude decrease. The changes (in percentage) of the peak-to-peak amplitude decrease were plotted as time-course curves.

### 4.4. Experiment 3: Effect of Diet and N-Acetylcysteine on Body Weight Gain

It has been known for years that a HFD results in excessive body weight gain in laboratory rodents [62,63] and in humans [64,65]. The aim of this experimental series was to study whether N-acetylcysteine treatment co-administered with HFD-priming could lead to lower body weight gain in young rats’ pups, as compared with control rats’ pups fed RD. The experiment was performed in separate series of naïve mice. Briefly, four groups of mice (n = 6, each group) were weaned 21 days postpartum and then fed a chow diet for one week. Afterwards, at day 28 of age, they were either subjected to RD (two groups) or HFD (two groups) for 2 weeks, with or without concomitant N-acetylcysteine co-administration in drinking water, as follows: Group 1, RD + N-acetylcysteine; Group 2: RD + N-acetylcysteine-free water; Group 3, HFD + N-acetylcysteine; Group 4: HFD + N-acetylcysteine-free water. In groups 1 and 3, 2 g/L N-acetylcysteine were co-administered in drinking water throughout the 2-week period of dietary treatment, according to the protocol of Ma et al. [34], while groups 2 and 4 received N-acetylcysteine-free water. The body weight of mice was assessed daily. In total, 12 RD-fed and 12 HFD-fed mice were used in this experiment.

### 4.5. Statistical Analysis

Single comparisons between two groups were analyzed by unpaired, two-tailed Student’s *t*- test. Time course experiments (peak-to-peak amplitude of NAc field potentials and mice body weight, along time) were analyzed by two-way repeated measures ANOVA followed by post hoc Bonferroni’s test for multiple comparisons. A 95% confidence interval was chosen for statistical significance. All analyses were performed using the software Prism version 8.1.0 (GraphPad, San Diego, CA, USA).

## 5. Conclusions

*Ad libitum* HFD consumption for a short 2-week period in young mice, followed by 5 days of HFD abstinence, resulted in upregulated levels of ΔFosB in the NAc core, together with suppression of LTD triggered in the NAc core from the mPFC. N-acetylcysteine administered 2.5 h before electrophysiology prevented LTD suppression observed in HFD-primed mice. Finally, body weight of HFD-fed mice was significantly higher than RD-fed, but this effect of HFD feeding on body weight was partially counteracted by co-administration of N-acetylcysteine in drinking water. Altogether, the above results: (i) highlight the hypothesis that *ad libitum* high-fat diet can trigger an addiction-like behavior, (ii) support the idea that the obesity epidemic may well rest on *ad libitum* availability of high fat and palatable diets, and (iii) suggest that N-acetylcysteine-based therapy may constitute a useful therapeutic approach to the problem of obesity in young people. It can be concluded that systematic studies in clinical settings are worth conducting to test N-acetylcysteine as a possible treatment for eating disorders.

## Data Availability

Not applicable.
